# Challenging the anthropocentric emphasis on phenotypic testing in prokaryotic species descriptions: rip it up and start again

**DOI:** 10.3389/fgene.2015.00218

**Published:** 2015-06-17

**Authors:** Iain C. Sutcliffe

**Affiliations:** Applied Sciences, Faculty of Health and Life Sciences, Northumbria UniversityNewcastle upon Tyne, UK

**Keywords:** genomics, phylogeny, species, systematics, taxonomy

“The benefits of specialization are tempered by the possibility that specialized groups become isolated, resist innovation, and engage in destructive competitiveness” (*Specialized Science*, Casadevall and Fang 2014).

Prokaryotic systematics is a highly specialized field and yet has fundamental reach and significance given that it provides the framework and, importantly, the names which we use to describe most of the microbial world. Names are given to prokaryotic taxa under the jurisdiction of the International Code of Nomenclature of Prokaryotes (ICNP; Lapage et al., 1992) and the vast majority of papers in prokaryotic systematics are descriptions that name taxa, particularly novel species and genera. Indeed, this century has seen a significant growth in the number of prokaryotic species named (Tamames and Rosselló -Móra, 2012; Oren and Garrity, 2014), with ~900 new names for *Bacteria* and *Archaea* published (either validly or effectively) in 2014. Mostly this growth in taxonomic activity is in Asia, with declines elsewhere (Tamames and Rosselló -Móra, 2012; Oren and Garrity, 2014).

Despite this progress, prokaryotic systematics has become isolated from mainstream microbiology and resistant to innovation. Moreover, the greatest challenge facing prokaryotic systematists remains the sheer scale of microbial diversity. Practitioners have thus to resolve two contradictory challenges. On one hand, there is view that novel strains “should be characterized as comprehensively as possible” (Tindall et al., 2010). On the other, there is the goal of mapping a taxonomic landscape likely to include >10^6^ species of *Bacteria* and *Archaea* (Yarza et al., 2014). Our progress to date (<15,000 species validly named, i.e., <1.5%) indicates that it is simply not pragmatic to pursue the ideal of characterizing each “as comprehensively as possible.” The current custom and practice of extensive phenotypic and chemotypic characterization runs contrary to the suggestion that classification can be achieved by *minimal* standards (Sutcliffe et al., 2012; Whitman, 2015) and it should be emphasized that the focus of the ICNP is on nomenclatural matters and the deposition of type materials. It is striking that the recent “minimal” standards for description of novel bifidobacteria, lactobacilli and relatives (Mattarelli et al., 2014) stipulate 26 “required” characteristics be tested (some of them multiple tests such as fermentation assays)!

In resolving this contradiction, the central question facing prokaryotic systematists thus becomes “how much characterization is necessary to demonstrate the distinctiveness of a taxon”? Despite the some well-articulated calls for reform (Rosselló-Móra, 2012; Sutcliffe et al., 2012; Zhi et al., 2012; Sangal et al., 2014; Vandamme and Peeters, 2014; Rosselló-Móra and Amann, 2015; Thompson et al., 2015; Whitman, 2015), there has not yet been the necessary critical reappraisal by the broader taxonomic community of what we are doing and why we are doing it. Consequently, as evidenced by hundreds of papers per annum, prokaryotic systematics has descended into a strait-jacketed and formulaic field, with practitioners uncritically performing self-imposed checklists of phenotypic and chemotypic tests, described by Oren and Garrity (2014) as an “assembly line” mostly culminating in “uninteresting and dull” papers, a situation often reinforced by peer reviewers and/or editors who propagate the same dogmatic adherence to the formula. This approach is damaging the field in at least four regards.

Firstly, many standard phenotypic tests generate data of poor reproducibility and with unacceptable margins of error, which cannot be considered good scientific practice. For example, “wet lab” G+C content determination has recently been shown to be unreliable in comparison to deducing this value from suitable quality genome sequence data (Kim et al., 2015). The insistence on the need for retesting of known type strains in parallel with putative novel strains betrays a lack of confidence in the reproducibility of data that has accumulated in the literature: if a method is reliable and robust, why should it need repeating? Secondly, many conventional tests are not only unreliable but also uninformative and/or irrelevant, particularly where single strains are being characterized, as is the case in >70% of prokaryotic species descriptions (Sutcliffe et al., 2012; Tamames and Rosselló -Móra, 2012; Oren and Garrity, 2014). Good examples are sugar fermentations, which are commonly assayed using commercial test strips. However, such characteristics are frequently variable within taxa and thus the data are of limited value for prokaryotic species described based on single isolates. Chemotypes may be stable but are typically investigated using low resolution analytical approaches. For example, criticisms of polar lipid analysis by two-dimensional thin layer chromatography have been given previously (Sutcliffe et al., 2012). The persistent use of unnecessary and/or uninformative experimental approaches also highlights the third aspect of damage to the field: resources are diverted away from the adoption of better technologies, which is especially problematic in a field that already struggles for funding (e.g., see Hedlund et al., 2015). Fourthly, as a consequence of the current formula, most prokaryotic species descriptions are now mostly boring lists of trivial characteristics (e.g., arcane enzyme activities and substrate utilization profiles, generated primarily because they are available in commercial kits), greatly reducing the attractiveness of the work to those outside the specialism. This is likely to negatively impact on the recruitment of promising young scientists into the field. The formulaic nature of most of these studies has drained the joy from the science and rendered it a sterile “stamp collecting” exercise. How can we then hope to attract the next generation of scientists into such a moribund field?

So, what are the solutions? Firstly, it is clear beyond reasonable dispute that genome sequence data alone can provide sufficient data to allow the robust discrimination of distinct taxa, whether this is based on overall genome relatedness indices (OGRI) or on tailored subsets of sequences, including conserved proteins (Chun and Rainey, 2014; Qin et al., 2014; Rosselló-Móra and Amann, 2015; Whitman, 2015). Moreover, the cost of genome sequencing continues to fall and is now within reach of most laboratories, particularly as savings should be made by discontinuing unneeded phenotypic and chemotypic tests. Secondly, it is clear that reliable thresholds for OGRI and other sequence analysis methods can now be defined for various taxonomic levels, although care still has to be taken in the flexible implementation of these, informed by knowledge of the particular taxa (Qin et al., 2014; Yarza et al., 2014; Kim et al., 2015; Rosselló-Móra and Amann, 2015). Now is the time for prokaryotic systematists to make an enthusiastic commitment to adopting methods to define prokaryotic taxa based primarily on genotypic criteria. Given the data-rich nature of genome sequences, and the ready application of this data to provide a phylogenetic basis for prokaryotic taxonomy, the advocates of extensive phenotypic testing now appear locked in an era that is both historic (harking back to a time when it was easier to measure phenotype than genotype, a situation effectively reversed by developments in DNA sequencing) and inherently anthropocentric: taking an experimental approach focussed on the limited number of parameters that we can easily measure (under the highly artificial conditions typically used for prokaryotic cultivation, including axenic culture) rather than deciphering those characters that are most relevant to the biology of the organism in its natural environment. In contrast, genome sequences provide data on the full complement of activities for which a strain is adapted, including the ca. 40% of genes present in any genome that encode proteins of unknown function, many of which may be highly significant *in situ*. Where multiple genome sequences are available, delineation of the core vs. accessory genome will provide reliable data on stable vs. variable characteristics of taxa. It is also notable that it is already possible (and will become increasingly straightforward) to predict metabolic capabilities and derive “*in silico* chemotaxonomy” from bioinformatic pathway analyses (Sutcliffe et al., 2012; Thompson et al., 2015). An excellent example is provided by *Catenulispora acidiphila*: originally reported to be not grow on cellulose, genome sequencing suggested the capacity to degrade cellulose, which was subsequently demonstrated experimentally (Anderson et al., 2012).

Crucial to driving the adoption of OGRI and related methods will be leadership from the *International Committee for Systematics of Prokaryotes* (ICSP), via a new *ad hoc* committee to provide authoritative guidance on the implementation of novel approaches (Sutcliffe et al., 2012, 2013), as has happened previously when disruptive technologies have led to step changes in taxonomic practice (Stackebrandt et al., 2002). The triumph of the implementation of the ICNP in the twentieth century has been to introduce an elegant and practical mechanism for governing prokaryotic nomenclature, bringing order and stability. The benefits and value of the ICNP are not in question. What instead needs challenging is dogmatic thinking over subjective interpretations that have created the impression that the ICNP is somehow “carved in stone.” Although a “2008 Revision” is expected shortly (Garrity, 2014), it is now timely to consider how the ICNP can be further adapted, particularly so as to allow minimal descriptions based on sequence data (Konstantinidis and Rosselló-Móra, 2015; Whitman, 2015).

The ICSP can also make an important contribution, in conjunction with the editors of key journals that publish descriptions of new prokaryotic taxa (notably the *International Journal of Systematic and Evolutionary Microbiology*), by introducing a streamlined publication format(s) for descriptions of prokaryotic taxa (Rosselló-Móra, 2012; Sutcliffe et al., 2012; Rosselló-Móra and Amann, 2015). A protolog format must be introduced that is database driven (machine readable), providing fields for: source and ecological details; minimal phenotypic data (either determined or deduced *in silico*), focussed on stable high-value characteristics rather than minutiae; supporting evidence that a taxon is distinctive (e.g., from OGRI or other sequence based methods); quality control information on the sequence data and links to sequence databases; details of accessibility of type materials; and sufficient etymological information to satisfy the requirements of the ICNP for valid naming. Such a format favors online publication with a short print summary, with names compiled in the Validation and Notification Lists and cross-referenced to databases such as LPSN (Parte, 2014). Moreover, because taxa can now be confidently and accurately delineated based on well-defined OGRI thresholds it seems likely that in many cases the consideration of new names for taxa could be made a matter of editor or curator oversight. This may have the benefit of removing the need for peer review of many proposals, given that finding appropriate peer reviewers is an increasing challenge due to the number of taxa being described annually. It is also envisaged that this format would be amenable to linking with databases deriving from other portable fingerprinting techniques, notably MALDI-TOF profiling (Schumann and Maier, 2014). Indeed, if polyphasic taxonomy is to have a future then it is likely to be through the integration of genomic data with portable fingerprint data such as that from mass spectrometry of proteins, lipids and/or metabolites.

Eventually a new *minimalist* protolog format could accommodate the formal naming of taxa not represented by cultivated strains but for which robust genomic sequence data is available, particularly if this is facilitated by a reappraisal of the criteria for use of the status *Candidatus* (Hedlund et al., 2015; Konstantinidis and Rosselló-Móra, 2015; Whitman, 2015). The traditional note or full paper formats would then be retained for cases where the delineation of novel taxa is not straightforwardly achieved by use of OGRI, or simply to serve author preference. Respecting the latter is consistent with Principle 1(4) of the ICNP, which protects freedom of taxonomic thought and action (it should be further emphasized that the ICNP only provides rules governing nomenclature and not taxonomic practice).

A new concise database driven format will have the benefit of permitting high-throughput accelerated naming of taxa, thereby placing landmarks on the taxonomic “map” of prokaryotic diversity (and bridging the worrying gulf between the small number of validly named taxa and the vast number of unnamed taxa). This would provide the platform from which future researchers can choose to revisit those taxa considered to be of particular interest, for more detailed characterization or to provide retrospective overarching analyses, including improved chemotyping using more advanced methodologies (for example, HPLC based peptidoglycan analysis, Desmarais et al., 2013; mass spectrometry in the case of lipids). The time and resources freed up by this new approach will allow prokaryotic systematists to address novel biological questions and challenges that go beyond the current mundane cataloging. As already exemplified by the pioneering Genomic Encyclopedia of Bacteria and Archaea project (Wu et al., 2009) and projects targeting the microbial “dark matter” (Hedlund et al., 2015), a genome based taxonomic “map” will better serve to guide the full spectrum of microbiological research from ecology and bioprospecting through to cell biology, genetics, and physiology.

The last two decades have seen significant progress in the naming of prokaryotic taxa and the development of molecular approaches for mapping microbial diversity. However, now is the time for another step change and it can be strongly recommended that best practice in prokaryotic systematics is descriptions of novel taxa based on genome sequence analyses of type strains. Where putative new taxa are indicated to be closely related (e.g., by preliminary 16S rRNA sequence analysis) to type strains lacking genome sequences, these should be determined in order to backfill the current gap between the total number of validly names species and those with genome sequenced type strains. Conservative reluctance to adopt genomics will damage the field greatly: to return to Casadevall and Fang (2014) “continuity and stability can also exclude new ideas and promote the phenomenon of groupthink, whereupon fields stagnate.” We cannot allow this to be the fate of prokaryotic systematics.

## Conflict of interest statement

The author is currently Editor-in-Chief of *Antonie van Leeuwenhoek* (Springer Publishing), which publishes significant numbers of prokaryotic species descriptions. He is writing in a personal capacity as an active prokaryotic systematist. The author declares that the research was conducted in the absence of any commercial or financial relationships that could be construed as a potential conflict of interest.
